# Impact of climate variability on *Plasmodium vivax* and *Plasmodium falciparum* malaria in Yunnan Province, China

**DOI:** 10.1186/1756-3305-6-357

**Published:** 2013-12-17

**Authors:** Yan Bi, Weiwei Yu, Wenbiao Hu, Hualiang Lin, Yuming Guo, Xiao-Nong Zhou, Shilu Tong

**Affiliations:** 1 School of Public Health and Social Work, Institute of Health and Biomedical Innovation, Queensland University of Technology, Victoria Park Rd, Kelvin Grove, Brisbane, QLD 4059, Australia; 2 Yunnan Center for Disease Control and Prevention, Kunming, China; 3 Guangdong Provincial Institute of Public Health, Guangdong Provincial Center for Disease Control and Prevention, Guangzhou, China; 4 School of Population Health, University of Queensland, Brisbane, Australia; 5 National Institute of Parasitic Diseases, Chinese Center for Disease Control and Prevention, Shanghai, China

**Keywords:** *Plasmodium vivax*, *Plasmodium falciparum*, Spatial cluster area, Distributed lag nonlinear model, Climatic variables, China–myanmar border area

## Abstract

**Background:**

Malaria remains a public health problem in the remote and poor area of Yunnan Province, China. Yunnan faces an increasing risk of imported malaria infections from Mekong river neighboring countries. This study aimed to identify the high risk area of malaria transmission in Yunnan Province, and to estimate the effects of climatic variability on the transmission of *Plasmodium vivax* and *Plasmodium falciparum* in the identified area*.*

**Methods:**

We identified spatial clusters of malaria cases using spatial cluster analysis at a county level in Yunnan Province, 2005–2010, and estimated the weekly effects of climatic factors on *P. vivax* and *P. falciparum* based on a dataset of daily malaria cases and climatic variables. A distributed lag nonlinear model was used to estimate the impact of temperature, relative humidity and rainfall up to 10–week lags on both types of malaria parasite after adjusting for seasonal and long-term effects.

**Results:**

The primary cluster area was identified along the China–Myanmar border in western Yunnan. A 1°C increase in minimum temperature was associated with a lag 4 to 9 weeks relative risk (RR), with the highest effect at lag 7 weeks for *P. vivax* (RR = 1.03; 95% CI, 1.01, 1.05) and 6 weeks for *P. falciparum* (RR = 1.07; 95% CI, 1.04, 1.11); a 10-mm increment in rainfall was associated with RRs of lags 2-4 weeks and 9-10 weeks, with the highest effect at 3 weeks for both *P. vivax* (RR = 1.03; 95% CI, 1.01, 1.04) and *P. falciparum* (RR = 1.04; 95% CI, 1.01, 1.06); and the RRs with a 10% rise in relative humidity were significant from lag 3 to 8 weeks with the highest RR of 1.24 (95% CI, 1.10, 1.41) for *P. vivax* at 5-week lag.

**Conclusions:**

Our findings suggest that the China–Myanmar border is a high risk area for malaria transmission. Climatic factors appeared to be among major determinants of malaria transmission in this area. The estimated lag effects for the association between temperature and malaria are consistent with the life cycles of both mosquito vector and malaria parasite. These findings will be useful for malaria surveillance–response systems in the Mekong river region.

## Background

Malaria, a wide-spread mosquito-borne disease, affects 106 countries around the world [1]. It is one of the leading causes of morbidity and mortality in many developing countries, responsible for about 216 million cases and approximately 665,000 deaths globally in 2011, with the majority, approximately 86% of malaria deaths occurring in children under five years of age [1]. In China, malaria remains a major public health issue, with 205,864 confirmed and 395,837 suspected cases and 158 reported deaths from 2005 to 2010, despite national malaria control efforts and international support in the past decades [2-9]. The endemic situation is even worse in the remote and poor border area of southern China [10,11]. For example, in Yunnan province, malaria incidence was 49.5/100,000 in 2005, with 15,072 confirmed and 26,084 suspected malaria cases and 38 reported deaths [4]. More than 11,000 people suffered from malaria each year from 2002 to 2006 with the province having the highest number of malaria cases and deaths in China for more than a decade since the 1990s [4]. There are frequent malaria outbreaks in Yunnan, which has devastating effects on mental, physical, social and economic development of individuals and villages affected [11,12].

A variety of factors are known to affect the transmission of malaria [13-17]. In particular, climatic factors are considered to play an important role in the spatial and temporal distribution of malaria [18,19]. The relationship between climatic variables and malaria transmission has been reported in many countries, mostly in Africa [15,20-22], Asia [2,3,23], South America and Latin America [24,25]. Malaria has been identified as one of the most climate sensitive diseases [26] with studies suggesting significant associations between temperature and malaria incidence [3,23,27,28]. Relative humidity [28-30] and rainfall [18,25] have also been associated with malaria transmission.

However, few studies have been conducted to examine the impact of climatic variables on malaria transmission in high risk areas along the border area of Yunnan Province [29,31,32], particularly in the Mekong river region. The Mekong neighboring countries share similar climatic and environmental conditions, and suffer from increasing risk of imported malaria by inter-country population movements. The Asian tropic zone is well known as a malaria endemic region [33], and Yunnan province is located in this zone, which shares a 4060 kilometer border with Myanmar, Laos and Vietnam. In 2005, the number of malaria cases in Yunnan accounted for more than one third (15,072/42,319) of the total cases in China [4], Yunnan faces a serious problem of malaria becoming endemic, particularly due to imported malaria along its border area [4]. 67% of *Plasmodium falciparum* cases (454/678) were imported from neighbouring countries in Yunnan in 2008, where the mobile population are vulnerable to malaria infection [10].

It is important to identify high risk areas of malaria transmission at the regional level, and to focus further on the micro level to assist determining risk factors and developing the optimal strategies for local malaria control and prevention. The spatial scan statistic method has been widely applied to identify cluster regions and periods in malaria transmission [34-37]. The distributed lag nonlinear model (DLNM) is a flexible model to show different delayed effects of the non-linear exposure–response relationship [38], and is increasingly used to examine the effects of temperature, rainfall and relative humidity on malaria transmission [23,39]. This study aimed to identify a high risk area of malaria at a county level in Yunnan Province, and to examine the effects of climatic variability on the transmission of *Plasmodium vivax* (*P. vivax*) and *Plasmodium falciparum (P. falciparum)* in the high risk area of Yunnan Province, China.

## Methods

### Study area

Yunnan Province has an area of 394,000 square kilometers and a population of 45.9 million according to the 2010 census. The province includes 16 prefectures, 128 counties and over 1,500 townships. The annual rainfall is about 1,100 mm, the seasonal mean temperature varies from 10°C to 15°C, and daily mean temperatures range from 12°C to 20°C. A bimodal epidemic pattern has been reported in Yunnan Province with the first peak in May–July and the second peak in October–November [35]. *P. vivax* and *P. falciparum* have been observed to co-exist in the province, with *P. vivax* being the dominant species. *An. sinensis* and *An. minimus* are two principal vector mosquitoes of malaria in Yunnan. *An. sinensis* is the dominant species and recognized as the primary vector in this region [11].

### Malaria cases

In China, malaria is a notifiable infectious disease through the National Notifiable Disease Report System (NNDRS). Daily malaria cases and deaths are reported to the China Center for Disease Control and Prevention (China CDC) based on township, county, prefectural, provincial and national levels since 2005. These cases and deaths were identified according to the unified diagnostic criteria issued by Chinese Ministry of Health [40]. Our investigations targeted Yunnan Province, which contains malaria high-risk border areas of southern China. In this study, malaria cases included parasite-based diagnosis (microscope and/or rapid diagnostic test), clinical diagnosis, suspected and parasite carrier, which was based on diagnostic criteria: patient’s travel history (i.e. stay/travel to endemic malaria area), symptoms (i.e. fever, chills and sweats), and laboratory testing (i.e. blood smear)/rapid diagnostic test [40]. The Giemsa staining method was used to test *plasmodium* parasites. We obtained information on daily malaria cases at a county level from the Yunnan Center for Disease Control and Prevention for the period from 1 January, 2005 to 31 December, 2010, including information about the date of onset, place of residence, type of parasite/s. Weekly *P. vivax* and *P. falciparum* malaria counts were calculated from daily records. A total of 44,877 malaria cases were reported during the study period. Eight records with missing birth dates and seven records with the reported dates in December 2004 were excluded. We excluded a further 948 cases (948/44884 = 2.1%) as their residence was not in Yunnan Province during the study period. A total of 41,578 malaria cases (including 31,704 *P. vivax* and 9,874 *P. falciparum*) with completed data for all variables were analysed in this study. Demographic data of each county were obtained from the annual book of the Yunnan Bureau of Statistics.

### Climate data

Temperature, relative humidity and rainfall data from 1 January, 2005 to 31 December, 2010 were obtained from the Chinese Meteorological Administration (http://www.cma.gov.cn). There are 36 weather stations in Yunnan province, and daily climate data are available in each station. In this study, two weather stations (Tengchong and Baoshan) were located at the identified high risk area of malaria transmission. The daily averaged climate data in these two stations were used to explore the relationship between climate variations and malaria. Weekly average values of minimum, maximum and mean temperatures, relative humidity and the weekly total rainfall were calculated from the daily data.

### Data analysis

Spearman’s correlation between weekly climatic variables (temperatures, relative humidity and rainfall) and malaria cases (*P. vivax* and *P. falciparum*) was examined using SAS 9.2 (SAS Institute Inc., Cary, NC, USA) to analyse bivariate relationships between two types of malaria parasite and potential climatic factors.

Spatial cluster analysis was conducted using Spatial Scan Statistics (SaTScan software, version 9.1, Martin Kulldorff, Boston, MA). A discrete Poisson model was used to identify purely spatial clusters of malaria incidence, for each county of Yunnan Province by year between 2005 and 2010. The cluster analysis was used to identify high risk areas in this study.

A Poisson regression model combined with distributed lag non-linear model (DLNM) was used to examine the effects of temperature, relative humidity and rainfall on the number of malaria cases as follows. The lag effects of climatic variables on numbers of *P. vivax* or *P. falciparum* malaria were separately examined.

First, the temperature indicator was selected by a comparison of Akaike’s Information Criterion (AIC) values for the corresponding models of mean, minimum and maximum temperatures. Minimum temperature was found to be a better predictor and was thus used as the temperature indicator.

Second, the cross-basis framework was built with natural cubic splines for temperature (degrees of freedom (*df* = 5)) and its lag (*df* = 5) along with relative humidity (*df* = 4) and its lag (*df* = 4). A polynomial function was used for rainfall (*df* = 4) and its lag (*df* = 4). The maximum lag was selected up to 10 weeks for all variables according to previous work (Kim *et al.*[23]).

Third, the Poisson regression model was applied:

$$\mathrm{Ln}\left(E\left(Y_{t}\right)\right)=\alpha +\sum_{i=1}^{p}\left(\beta_{i}x_{i}\right)+\mu_{j}\mathrm{seaso}n_{j}+s\left(w_{j},7\right)$$

Where *t* refers to the week of the observation; (*Y*_*t*_) denotes the observed weekly malaria counts on week t; *x*_*i*_ denotes the cross-basis of temperature and relative humidity when their effects on malaria were examined, and rainfall and temperature when the effects of rainfall on malaria were examined; season_*j*_ denotes seasonal effects that were controlled by a categorical variable consisting of dry (November–April) or wet (May–October) season; *w*_*j*_ is the week of the year *j* (e.g. 1, 2,…,52*)* and natural cubic splines with 7 degrees of freedom were used to adjust for seasonal and long-term effects; α is the intercept term; p is the number of variables; *β a*nd *μ* are coefficients.

Fourth, we plotted the associations of minimum temperature, relative humidity and rainfall with malaria cases for both parasites. The central value for each climatic variable was identified by the visual inspection of the plots.

Fifth, we repeated the second and third steps with central values of minimum temperature (7°C), relative humidity (73%) and rainfall (60-mm), which was identified by step four.

Finally, parasite-specific risk estimates of weekly malaria cases associated with one unit increase in temperature, relative humidity and rainfall above the central value over different lags were evaluated.

The residuals were checked to evaluate the adequacy of the model. Sensitivity analyses were performed to make sure that the associations between climate variables and malaria did not change substantially when the degrees of freedom for climate variables changed. All data analyses were conducted using “dlnm” functions of R packages to fit the regression model (The R Foundation for Statistical Computing, version 2.15.2, 2012 http://cran.ms.unimelb. edu.au/).

### Ethical approval

An ethical approval was granted by the Queensland University of Technology (N0.1000000573). A statement of filed data collection was provided by the Yunnan Center for Disease Control and Prevention, China.

## Results

### Exploratory analyses

Table 1 shows the summary statistics of weekly *P. vivax* and *P. falciparum* and climatic variables in the identified primary cluster area of western Yunnan. During the study period, the weekly mean malaria cases were 60.8 for *P. vivax* and 25.3 for *P. falciparum,* respectively. In the total 313 weeks of the study period, there were 27,052 malaria cases (including 19,106 *P. vivax* and 7,946 *P. falciparum*) reported in the identified high risk cluster area. The average values of the maximum, mean and minimum temperatures were 25°C, 18.5°C and 14.3°C, respectively. The average weekly relative humidity and rainfall were 73.1% and 27.3 mm, respectively.

**Table 1:** Summary statistics of weekly malaria cases and weather conditions in the primary cluster area of Yunnan Province, China, 2005–2010

**Variables**	**Mean (SD)**	**Minimum**	**Percentile**			**Maximum**
			**25%**	**50%**	**75%**	
Temperature (°C)						
Maximum	25.0 (3.4)	8.6	22.8	25.8	27.5	31.3
Mean	18.5 (4.3)	5.3	14.8	20.1	22.1	24.9
Minimum	14.3 (5.5)	2.1	9.0	15.4	19.7	21.6
Relative humidity (%)	73.1 (10)	32.6	66.3	75.2	81	91.9
Rainfall (mm)	27.3 (32.2)	0	0.31	16.2	43.9	156.3
Plasmodium parasite						
*P.v*	60.8 (50.3)	5	21	41	96	210
*P.f*	25.3 (24.6)	0	8	15	34	132

*P.v: Plasmodium vivax*, *P.f*: *Plasmodium falciparum*.

Weekly numbers of malaria cases (*P. vivax* and *P. falciparum*) were associated with weather variables. Temperature and rainfall positively correlated with both *P. vivax* and *P. falciparum*, while, relative humidity was positively associated with *P. vivax* but not *P .falciparum*. The three temperature indicators were strongly correlated with each other (Table 2). We used minimum temperature in the subsequent analyses, because it gives the best model fit.

**Table 2:** Spearman correlation coefficients between weekly malaria cases and weather variables in the high risk area of Yunnan, China, 2005– 2010

**Variables**	**Maximum temperature**	**Mean temperature**	**Minimum temperature**	**Relative humidity**	**Rainfall**	***P. v***
Mean temperature	0.87*					
Minimum temperature	0.72*	0.96*				
Relative humidity	0.07*	0.48*	0.67*			
Rain (weekly cumulation)	0.40*	0.70*	0.80*	0.73*		
*P. v*	0.37*	0.46*	0.45*	0.35*	0.33*	
*P. f*	0.32*	0.40*	0.39*	0.30*	0.30*	0.89*

*p < 0.001.

**Figure 1:**
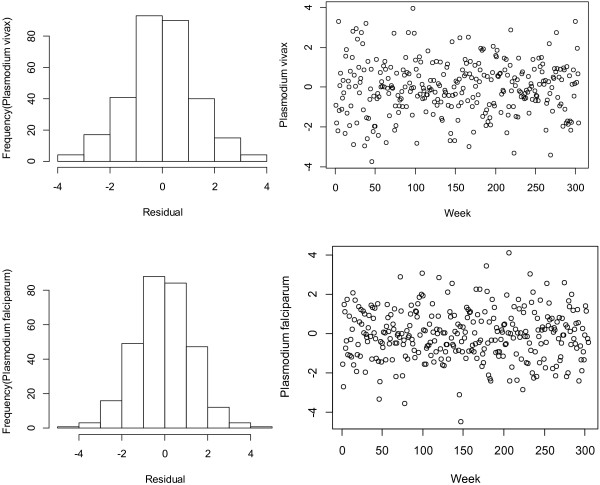
**Checking the residuals for the temperature–malaria model.** The left side histogram of residuals. The right side scatter plot of residuals over time.

**Figure 2:**
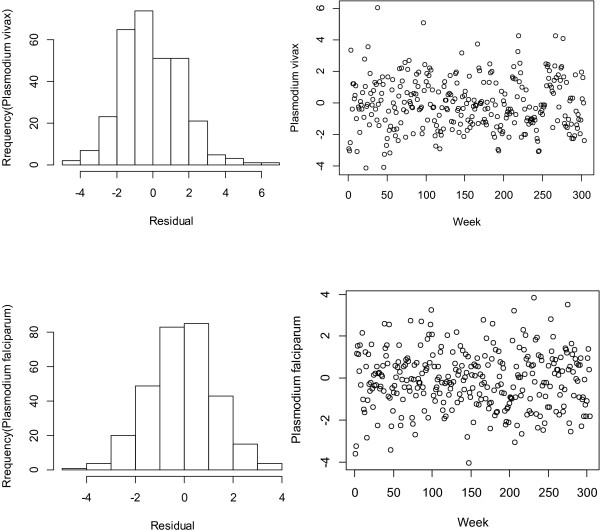
**Checking the residuals for the rainfall–malaria model.** The left side histogram of residuals. The right side scatter plot of residuals over time.

The residuals were checked to evaluate the adequacy of the model to ensure they were approximately normally distributed and independent over time (Figures 1 and 2). A sensitivity analysis was conducted by changing the *df* for temperature (3–7), relative humidity (3–7), rainfall (3–7) and week of the year (6-15). No substantial changes were found. We changed the *df* (6-15) to control for the week of the year, and the results varied slightly.

### Spatial analysis

**Figure 3:**
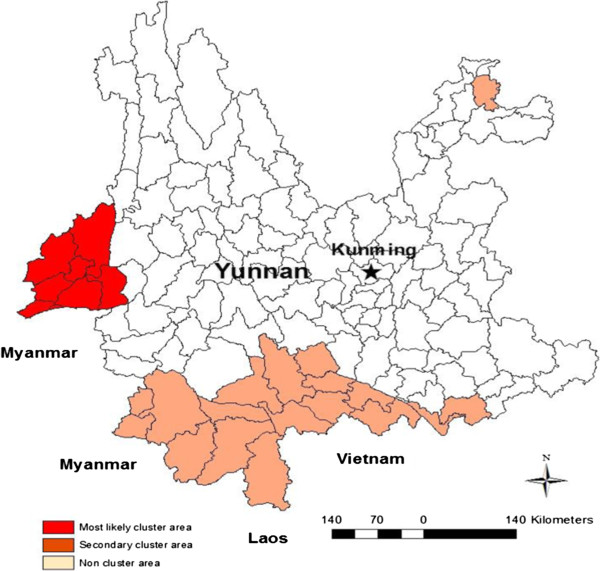
**Study area: Yunnan Province, China [using ArcGIS software, version 10.0, USA, Environmental Systems Research Institute].** The primary study area (in red) is the most likely cluster area which is located in the western part of Yunnan Province. Malaria cases in 8 primary cluster counties contributing to > 60% (27,052/43,929) of the total cases for Yunnan from 2005 to 2010.

Spatial Scan Statistics identified a most likely cluster of malaria cases including eight counties (Relative Risk (RR) = 36.10, P < 0.01) in western Yunnan and secondary clusters encompassed 18 counties, with significant relative risks between 1.52 and 4.11 from 17 counties in southern Yunnan and one in North eastern Yunnan (Figure 3). Additional time series analysis was conducted in the most likely cluster area with eight clustering counties bordering Myanmar locating in a subtropical region in western Yunnan.

### Estimated effects of climate variables on *P. vivax* and *P. falciparum*

**Figure 4:**
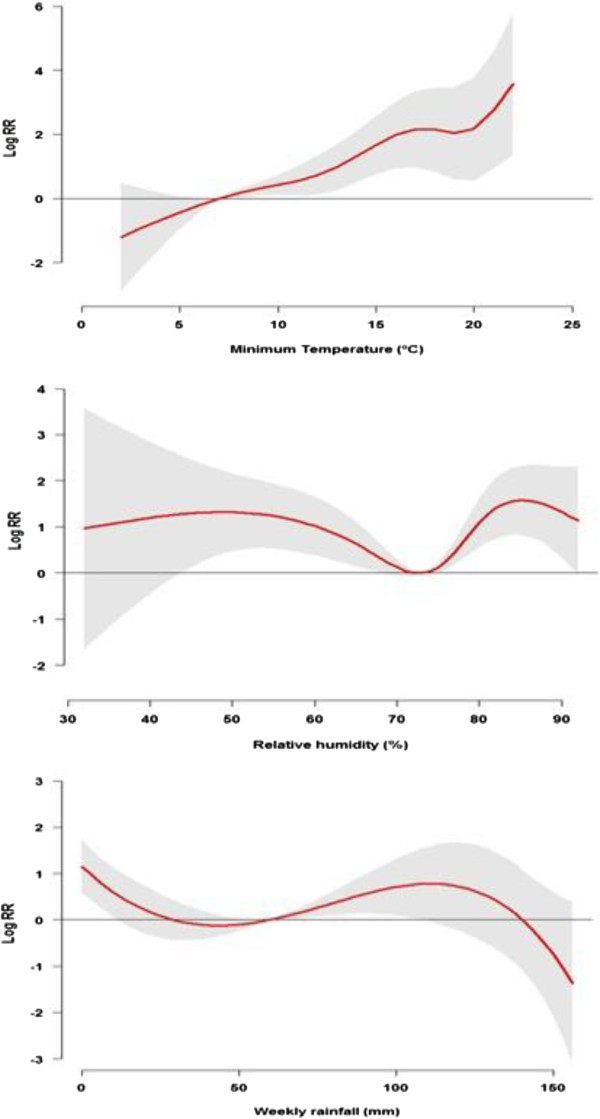
**The estimated overall effects of minimum temperature, relative humidity and rainfall on *Plasmodium vivax *along the lag of 0–10 weeks in the high risk area of Yunnan, China, 2005–2010.** (The red lines are mean relative risks, and grey regions are 95% confidence intervals).

**Figure 5:**
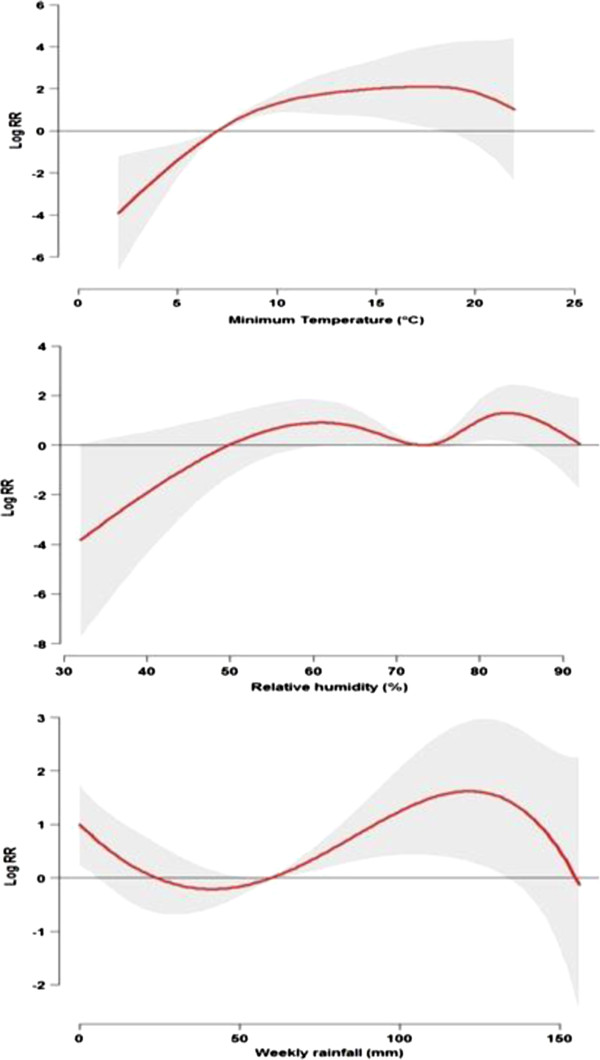
**The estimated overall effects of minimum temperature, relative humidity and rainfall on *Plasmodium falciparum *along the lag of 0–10 weeks in the high risk area of Yunnan, China, 2005–2010.** (The red lines are mean relative risks, and grey regions are 95% confidence intervals).

Table 3 shows estimated single-week and overall lag effects of minimum temperature, relative humidity and rainfall on *P. vivax* and *P. falciparum*. The effects of minimum temperature, relative humidity and rainfall on *P. vivax* (Figure 4) and *P. falciparum* (Figure 5) were estimated by 1°C rise in minimum temperature with 7°C as a reference, by 10% rise in relative humidity with 73% as a reference and by 10-mm rise in rainfall with 60-mm as a reference.

**Table 3:** **The weekly lag effects of climate variables on *P. vivax *** **and *P. falciparum***

**Lag effects on *P. v***	**Minimum temperature (°C)**	**Relative humidity (%)**	**Rainfall (mm)**
Lag0	1.01 (0.96,1.06)	1.08 (0.85,1.38)	0.99 (0.97,1.01)
Lag1	0.98 (0.94,1.03)	1.08 (0.93,1.27)	1.02 (0.99,1.04)
Lag2	0.99 (0.96,1.02)	1.12 (0.95,1.33)	1.03 (1.01,1.05)*
Lag3	1.01 (0.98,1.04)	1.18 (1.05, 1.33)*	1.03 (1.01,1.04)*
Lag4	1.02 (1.00,1.04)*	1.23 (1.10,1.38)*	1.02 (1.00,1.04)*
Lag5	1.03 (1.01,1.05)*	1.24 (1.10,1.41)*	1.02 (0.99,1.05)
Lag6	1.03 (1.01,1.06)*	1.23 (1.09,1.39)*	1.01 (0.99,1.02)
Lag7	1.03 (1.02,1.05)*	1.19 (1.07,1.33)*	1.01 (0.99,1.02)
Lag8	1.03 (1.01,1.05)*	1.13 (1.02,1.27)*	1.01 (0.99,1.03)
Lag9	1.02 (1.00,1.04) *	1.07 (0.93,1.25)	1.02 (1.00,1.04)*
Lag10	1.0 (0.98,1.05)	1.02 (0.83,1.25)	1.02 (1.00,1.05)*
Lag0-10	1.19 (1.04,1.35)*	4.38 (1.86,10.30)*	1.18 (1.05,1.34)*
Lag effects on *P. f*			
Lag0	1.02 (0.95,1.10)	0.95 (0.55,1.62)	0.98 (0.96,1.01)
Lag1	1.06 (0.99,1.14)	0.87 (0.60,1.26)	1.02 (0.99,1.05)
Lag2	1.05 (0.99,1.10)	0.89 (0.60,1.33)	1.04 (1.01,1.06)*
Lag3	1.05 (0.99,1.10)	0.98 (0.74,1.30)	1.04 (1.01,1.06)*
Lag4	1.06 (1.03,1.09)*	1.07 (0.82,1.39)	1.03 (1.01,1.06)*
Lag5	1.07 (1.04,1.11)*	1.11 (0.82,1.51)	1.02 (0.99,1.05)
Lag6	1.07 (1.04,1.11)*	1.12 (0.83,1.51)	1.01 (0.99,1.04)
Lag7	1.07 (1.03,1.11)*	1.10 ( 0.84,1.43)	1.01 (0.98,1.03)
Lag8	1.06 (1.03,1.09)*	1.06 (0.82,1.37)	1.01 (0.98,1.04)
Lag9	1.04 (1.01,1.08)*	1.00 (0.71,1.41)	1.01 (0.99,1.04)
Lag10	1.02 (0.97,1.08)	0.94 (0.57,1.54)	1.03 (0.99,1.06)
Lag0-10	1.74 (1.41,2.16)*	1.06 (0.16,6.78)	1.23 (1.03,1.46)*

*P < 0.05.

For *P. vivax,* significant effects of temperature appeared at a lag of 4 weeks. The effects persisted up to 9 weeks. The RR for a 1°C increase in minimum temperature was associated with 1.02 (95% CI, 1.00, 1.03) at a 4–week lag, and with a maximum RR of 1.03 (95% CI, 1.01, 1.05) at a lag of 5 weeks. Significant effects of relative humidity and rainfall were observed at a lag of 3–8 weeks for relative humidity, and a short lag of 2–4 weeks and a long lag of 9-10 weeks for rainfall, respectively. The RR with a 10% increment in humidity was associated with 1.18 (95% CI, 1.05, 1.33) at a lag of 3 weeks, and a 10-mm increment in rainfall was associated with 1.03 (95% CI, 1.01, 1.05) at a lag of 2 weeks. The overall effects (lag 0–10 weeks) of temperature, relative humidity and rainfall on *P. vivax* cases were significantly observed, with the RRs of 1.19 (95% CI, 1.04, 1.35) for temperature, 4.38 (95% CI, 1.86, 10.30) for relative humidity and 1.18 (95% CI, 1.05, 1.34) for rainfall, respectively (Table 3).

For *P. falciparum,* significant effects of temperature were observed at lags of 4–9 weeks and overall 0–10 weeks. Compared to *P. vivax,* the minimum temperature had a larger effect on *P. falciparum*. The RR for a 1°C increase in minimum temperature was associated with 1.06 (95% CI, 1.03, 1.09) at a lag of 4 weeks and 1.74% (95% CI, 1.41, 2.16) at a lag of 0–10 weeks, respectively. *P. falciparum* malaria cases were significantly associated with a 10-mm increase in weekly rainfall at lags of 2–4 weeks and overall 0–10 weeks with similar magnitude as *P. vivax*. A significant association between humidity and *P. falciparum* was not observed at any lags (Table 3).

## Discussion

In this study, we identified a high risk area of malaria transmission in Yunnan Province, southern China, during 2005–2010. We also examined the effects of weekly minimum temperature, relative humidity and rainfall on the transmission of *P. vivax* and *P. falciparum* in this area*.* The results of the study showed that the primary cluster area included eight counties in western Yunnan along the China–Myanmar border. Minimum temperature, relative humidity and rainfall were significantly associated with the transmission of *P. vivax* and *P. falciparum*, which provided detailed weekly information on the relationship between climatic factors and malaria transmission in the border area of Yunnan, China. These findings are vital for planning and implementing local malaria interventions, and contribute to strategy development for controlling and eliminating “border malaria” in Mekong river regional countries [41].

### Identified high risk areas

Spatial cluster analysis can provide a public health tool for investigating high risk areas and clues for possible disease risk factors [3]. The identified malaria high risk area in this study, which located in the subtropical region along the China–Myanmar border was consistent with the findings of previous studies [3,35,37]. Malaria transmission in the border area of Yunnan has been an important issue in China due to the increasing malaria infections imported from neighbouring countries. The imported malaria cases are regarded to play a key role in initiating frequent outbreaks in Yunnan border [4,11]. Relatively high transmission mainly occurs in this bordering region compared to other provinces in China [42]. Among three China–Myanmar, China–Laos and China–Vietnam border areas in Yunnan Province, China-Myanmar has the longest border (1997 km), the highest annual parasite incidence rate (API, > 2.3%) and the highest proportion of *P. falciparum* reported in positive blood smears (33%) [11]. This bordering region is a stable *P. falciparum* transmission area [2]. The identified high risk area along the China–Myanmar border in western Yunnan appeared to be the dominating source of *P. falciparum* imported to other 21 provinces among 23 provinces reporting malaria cases in mainland China [2,8,37]. Although *P. falciparum* contributed to the majority of the imported cases in China [4], the current study found that there were more *P. vivax* malaria cases than *P. falciparum* malaria cases along the China–Myanmar border, with the ratio of *P. vivax* to *P. falciparum* being 2.4:1 (19,106:7,946). The results were contrary to the situation along the Thailand–Myanmar border where *P. falciparum* was a dominant malaria species [31], Thus, the local policy for malaria intervention should prioritise both parasites for bordering areas in Mekong river regional countries.

### Estimated effects of temperature and relative humidity on malaria

Both temperature and relative humidity are considered environmental risk factors for malaria transmission [42]. In this study, significant associations between minimum temperature and the number of malaria cases appeared at a lag of 4 weeks, and up to 9 weeks for both types of malaria parasites after adjusting for relative humidity. An effect of maximum temperature for a lag of 3 weeks on *P. vivax* was reported in a temperate area in the Republic of Korea (ROK) [23]. Other previous studies used monthly data and found that a significant association between minimum temperature and malaria transmission at a lag of one to two months [43-45]. Temperature plays a crucial role in the transmission cycle of malaria parasite and mosquito survival [46]. Studies found that at the temperature of 22°C, a life cycle of malaria parasite development in mosquito vector is completed at less than 3 weeks [47-49]. If the humidity remains between 55% and 80%, it takes 15–25 days for a *P. vivax* vector to complete its life cycle when the temperature varies from 15–20°C, and 20–30 days for a *P. falciparum* vector to complete its life cycle if the temperature varies between 20–25°C [50]. In the study area, the lag effect started from week four and lasted to week nine, with a minimum temperature range of 11–16°C, which is consistent with the time required for development of mosquito vector and for completion of the parasite life cycles in the local vector mosquito. An outbreak of the infection could therefore be predicted from climate forecasts, allowing early warning to be given. A climate-based early warning system could be used in this area to alert the authorities of possible changes in the risk level, either immediately or in the near future and to take action to protect the vulnerable members of the population.

Relative humidity seems to have an indirect effect on not only the development of parasites but also the activity and survival of anopheline mosquitoes [51]. Relative humidity has been found to be one of the key determinants for the transmission of malaria, with low humidity observed to limit the distribution and abundance of mosquito vectors in China [52]. A recent study of the impacts of climate change on malaria in China classified three malaria transmission zones based on monthly temperature and relative humidity changes, with Yunnan found to be located in a high risk zone of malaria transmission [42]. Our study identified a hot spot of malaria transmission in this high risk zone and narrowed it down to a local county scope. According to our estimates, the minimum temperature and humidity have significant effects on malaria, with the range of relative risk from 1.02 to 1.03 on *P. vivax*, and from 1.04 to 1.07 on *P. falciparum* for a 1°C increase in minimum temperature, and from 1.02 to 1.04 on *P. vivax,* for a 10% increase in relative humidity. An association between relative humidity and *P. falciparum* was not found in this subtropical area, which is consistent with a previous study carried out in a tropical rain forest area along the China–Laos border in southern Yunnan [45]. This may be due to the fact that the majority of *P. falciparum* cases are imported infections from neighbouring countries [4], and therefore relative humidity is probably not a limiting factor for *P. falciparum* in this area [45]. These findings provide important information for early intervention initiatives and in developing strategies for local malaria surveillance–response systems.

### Estimated effects of rainfall on malaria

Rainfall is considered to be the predominant climatic factor on the transmission of malaria [53]. Rainfall is found to have a great influence on the completion of the life cycle of the malaria parasite [39] and it modifies the effects of temperature and increases the effects of humidity [52]. However, the influence of rainfall on malaria transmission is complex. Our results show different lag effects of rainfall on *P. vivax* and *P. falciparum*, which provide an example to explain to some extent such a complex inter-relationship*.* Two stages of the lag effects were examined, with a short lag of 2–4 weeks and a long lag of 9–10 weeks for *P. vivax,* and a short lag of 2–4 weeks for *P. falciparum*. This pattern in effect may be due to rainfall increasing breeding sites for vector mosquitoes such as pools of water, with a short lag effect of 2–4 weeks supporting this hypothesis. By contrast, heavy rainfall may destroy existing breeding places, interrupt the development of mosquito eggs or larvae, or flush the eggs or larvae out of the pools [45]. Therefore, it will take longer time to rebuild the mosquito life cycle for an infection. In addition, evaporation of pools keeps relative humidity at a high level which prolongs longevity of vector mosquitoes, and may have resulted in the long lag effect of 9–10 weeks for *P. vivax*. A long lag effect on *P. falciparum* was not observed in our study*.* The reason could be that overseas-imported *P. falciparum* has begun to dominate and other factors (e.g. life cycle of parasite, parasite adaptation, mosquito habits, etc) might be different from *P. vivax*. Further research on this issue is warranted in the future.

### Strengths and limitations

There are three strengths in this study. Firstly, this is the first study to assess the relationship between climatic factors and malaria transmission in China–Myanmar border. Secondly, we obtained daily data of *P. vivax* and *P. falciparum* malaria cases, and daily weather data at a county level in Yunnan Province, China. This facilitated a detailed local assessment. Thirdly, we applied a DLNM to examine the weekly exposure–response relationship and its distributed lag effects [38] on two types of malaria parasite. These results provide valuable information for both local health authorities and neighboring countries.

Two limitations of this study should also be noted. Firstly, we only examined the effects of climatic variables on malaria transmission, but non-climatic factors, such as human activities, socioeconomic status, vector control programs, drug resistance and environmental changes, may also affect the spread of this disease [14,26,54,55]. However, these non-climatic factors are unlikely to vary significantly on a weekly scale and were unavailable for this research. Secondly, an investigation carried out in 2001 suggested that the under-reported malaria cases were five times higher than the actual number of reported cases in Yunnan [56]. The true number of malaria cases would therefore be much greater than that reported. Thus the association between climatic variables and malaria cases could be much stronger. The study results will have substantial implications for the allocation of health resources.

## Conclusion

In summary, a high risk area of malaria transmission was identified along the China–Myanmar subtropical region in Yunnan Province. The number of malaria cases in this high risk border area is largely dependent on climatic conditions including temperature, relative humidity and rainfall. The estimated lag effects for the association between temperature and malaria are consistent with the life cycles of both the mosquito vector and malaria parasite. The results of this study will be useful for malaria surveillance-response systems in the Mekong river region. These findings may also be applicable to countries with a similar problem of malaria transmission.

## Abbreviations

P. v: Plasmodium vivax; P. f: *Plasmodium falciparum*; An.: *Anopheles*; NNDRS: National Notifiable Diseases Report System; China CDC: China center for disease control and prevention; DLNM: Distributed lag nonlinear model; RR: Relative risk; AIC: Akaike’s information criterion; df: Degrees of freedom; 95% CI: 95 percent confidence interval.

## Competing interests

The authors declare that they have no competing interests.

## Authors’ contributions

ST and YB conceptualised this study and YB designed the research protocol and wrote the manuscript. YB and HL performed field data collection. WY, YB and YG conducted data management and analysis. ST, WY and WH contributed to interpret the results and assisted in writing the manuscript. XZ contributed to the manuscript by providing intellectual feedback on draft. All authors read and approved the final version of the manuscript.
